# Strategic intergroup alliances increase access to a contested resource in male bottlenose dolphins

**DOI:** 10.1073/pnas.2121723119

**Published:** 2022-08-29

**Authors:** Richard C. Connor, Michael Krützen, Simon J. Allen, William B. Sherwin, Stephanie L. King

**Affiliations:** ^a^Biology Department, University of Massachusetts Dartmouth, North Dartmouth, MA 02747;; ^b^Department of Biological Sciences, Marine Sciences Program, Florida International University, North Miami, FL 33181;; ^c^Evolutionary Genetics Group, Department of Anthropology, University of Zurich, CH-8057 Zurich, Switzerland;; ^d^School of Biological Sciences, University of Bristol, Bristol BS8 1TQ, United Kingdom;; ^e^School of Biological Sciences, University of Western Australia, Crawley, WA 6009, Australia;; ^f^Evolution and Ecology Research Centre, School of Biological, Earth and Environmental Sciences, University of New South Wales, Sydney, NSW 2052, Australia

**Keywords:** cooperation, social evolution, alliance formation, bottlenose dolphin

## Abstract

Efforts to understand human social evolution rely largely on comparisons with nonhuman primates. However, a population of bottlenose dolphins in Shark Bay, Western Australia, combines a chimpanzee-like fission-fusion grouping pattern, mating system, and life history with the only nonhuman example of strategic multilevel male alliances. Unrelated male dolphins form three alliance levels, or “orders”, in competition over females: both within-group alliances (i.e., first- and second-order) and between-group alliances (third-order), based on cooperation between two or more second-order alliances against other groups. Both sexes navigate an open society with a continuous mosaic of overlapping home ranges. Here, we use comprehensive association and consortship data to examine fine-scale alliance relationships among 121 adult males. This analysis reveals the largest nonhuman alliance network known, with highly differentiated relationships among individuals. Each male is connected, directly or indirectly, to every other male, including direct connections with adult males outside of their three-level alliance network. We further show that the duration with which males consort females is dependent upon being well connected with third-order allies, independently of the effect of their second-order alliance connections, i.e., alliances between groups increase access to a contested resource, thereby increasing reproductive success. Models of human social evolution traditionally link intergroup alliances to other divergent human traits, such as pair bonds, but our study reveals that intergroup male alliances can arise directly from a chimpanzee-like, promiscuous mating system without one-male units, pair bonds, or male parental care.

## Significance

Cooperation between allied individuals is ubiquitous in human societies. Our capacity to build strategic cooperative relationships across multiple social levels, such as trade or military alliances both nationally and internationally, is thought to be unique to our species. Here, however, we show that male bottlenose dolphins form the largest known multilevel alliance network outside humans, where the cooperative relationships between groups, rather than alliance size, increases male access to a contested resource. These results reveal that both dolphins and humans form strategic intergroup alliances between unrelated individuals, likely selecting for enhanced social cognition. This surprising case of convergence suggests that dolphin societies, as well as those of nonhuman primates, are valuable model systems for understanding human social and cognitive evolution.

Traditional human societies, such as hunter-gatherers, have a nested social structure, with the family group at the core that associates in multifamily groups, often called bands, which are linked in an ethnolinguistic group (1, 2). Although other primates have multilevel societies (3), humans (*Homo sapiens*) are considered unique in the extent to which the distinct levels of social structure are based on cooperative alliances (4–6). Indeed, Chapais described nested human social units as a “federation” (2), and in modern humans, the ability to form strategic cooperative relationships, or alliances, between groups extends to relationships even between nation states (7). To date, efforts to understand human social evolution have focused almost exclusively on comparisons with other primates, especially chimpanzees (*Pan troglodytes*) and baboons (*Papio* spp.) (8–11). However, key insights may also be gained by evaluating convergence between humans and more distantly related taxa.

The *bauplan* and habitat of dolphins could not be more unlike that of primates. It is surprising, therefore, that a population of Indo-Pacific bottlenose dolphins (*Tursiops aduncus*) in Shark Bay, Western Australia, has converged with humans in their formation of multilevel male cooperative alliances and with chimpanzees in their grouping patterns, mating system, and life history traits (12, 13). The male alliances in Shark Bay must be understood in the context of the population social structure, ecology, and, particularly, female reproductive tactics. There is no closed bisexual social unit, similar to a chimpanzee community or human band; rather, there is a continuous mosaic of male alliance ranges superimposed over a similar mosaic of generally smaller female ranges ((14, 15), *SI Appendix*). There is also no evidence of territoriality, seasonal or otherwise, and both sexes are philopatric (14). Female home ranges and grouping behavior in Shark Bay are highly variable and likely related to variation in learned foraging tactics (16–19).

Alliances are defined as enduring relationships with repeated instances of cooperation ((20), see *SI Appendix* for full operational definition of alliances), and the male dolphin alliance system in Shark Bay is driven by cooperation and competition over access to females, where two to three males (i.e., first-order alliances) form aggressively maintained consortships with individual females that last from hours to weeks. First-order alliances are therefore defined based on functional behavior, i.e., herding a female together. Almost all adult males are members of a second-order alliance, which comprises 4–14 members that compete with other alliances over females (14, 21, 22). First-order allies are chosen from within a male’s second-order alliance (22), and the stability of first-order alliances may be highly variable. For instance, some males have consistent first-order allies, while others will consort females with numerous males from within their second-order alliance in a given mating season (22–24). Although it is currently impossible to determine how matings are shared during consortships, males who have stronger and more homogenous social bonds within their second-order alliance obtain more paternities (25).

Second-order alliances are defined using both quantitative measures, i.e., hierarchical clustering analysis of association indices (26), and functional behavior, i.e., cooperating in the attempted theft and defense of females (21, 22). Second-order alliances form when males in their early to midteens strengthen bonds with mostly unrelated male associates, as determined via genetic data, of a similar age from their extended juvenile period (27, 28). Membership in second-order alliances is largely closed, as adults rarely join or leave to join other second-order alliances (reviewed in (22), *SI Appendix*). With gradual attrition due to death, second-order alliances can last for decades and are therefore considered the core male social unit in Shark Bay (22). Male dolphins in Shark Bay also form between-group alliances, i.e., third-order alliances, a rare phenomenon outside of humans, when second-order alliances associate preferentially and cooperate in contests over females (24, 29). Hostile interactions between second-order alliances over access to females are common, and the risk of injury for males during these physical altercations can be high (24). Given that second- and third-order alliances have the same function, that is cooperation against other groups over females, the value of third-order allies may lie in having a greater number of possible allies in proximity given the often-dispersed nature of second-order allies and the substantial variation in second-order alliance size (29). Third-order alliances are therefore defined by significant association preferences between second-order alliances (quantified using permutation tests (26)) and functional behavior (24, 29).

Social bond strengths within second-order alliances can be highly differentiated, and as a result, bond strengths between some third-order allies are comparable to those between members of the same second-order alliance (24). However, there are a number of factors that clearly distinguish second- from third-order alliances. First, separate association analyses of each third-order alliance show that males cluster within their second-order alliances (*SI Appendix*) and males are rarely observed with third-order allies without the presence of second-order allies (*SI Appendix*, Fig. S1). Second, males form first-order alliances and consort females almost exclusively with other males from within their second-order alliance ((22, 23), *SI Appendix*). Third, second-order alliances, once formed, are largely closed; it is rare for adult males to move to a different second-order alliance (*SI Appendix*). Fourth, playback experiments of individually distinctive identity signals (termed signature whistles) demonstrated that males categorize members of their second-order alliance as “team” members, independently of social bond strength across all three alliance levels (24). Finally, vocal exchanges between males can be used to maintain key social relationships and are significantly more likely to occur within second-order alliances than third-order alliances, which would not be the case if second- and third-order allies were interchangeable (30).

All three levels of male dolphin alliances exhibit characteristics that are associated with strategic alliance formation in other animal social groups, such as nonhuman primates. These include alliance formation among nonrelatives, highly differentiated alliance relationships, strategic temporary and long-term shifts in allegiance (what Nishida (31) called “allegiance fickleness”), and the use of affiliative contact behavior to form and maintain alliance bonds (22, 30, 32, 33). However, while previous studies have linked within alliance relationships with increased access to females and, subsequently, male paternity success (25), it remains unknown whether between-group alliances in dolphins, i.e., third-order alliances, also increase access to females. Here, we use comprehensive association and consortship data collected between 2001 and 2006 on 121 well-studied focal males (12 second-order alliances and five trios that remained from formerly larger second-order alliances) to extend our understanding of the strategic nature of dolphin multilevel alliances. We examine the extent of male dolphin social networks and how variation in social bonds, and especially bond strength at all three alliance levels, impacts male success. We show that (i) the Shark Bay dolphin alliance network is continuous and the largest known among nonhumans, (ii) that associations and variation in bond strength within alliances predict male cooperation and success (i.e., access to females), and (iii) as in humans (34, 35), between-group alliances are important for male success.

## Results

**Fig. 1.**
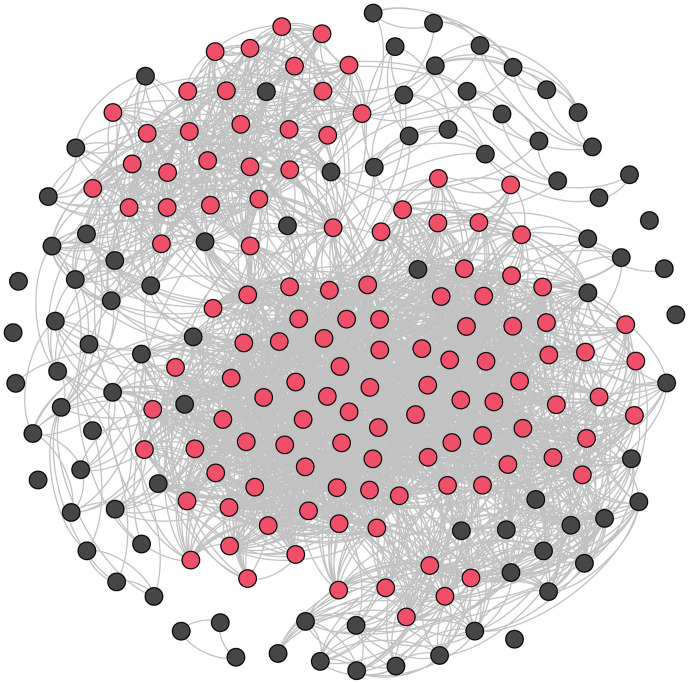
Male alliance association network in the eastern gulf of Shark Bay between 2001 and 2006 (*n* = 202). The network figure shows the well-studied focal males (121 of these in red) and nonfocal males (81 in black). The gray lines denote unweighted relationships (i.e., they show a relationship is present but do not represent the strength of the relationship). Associations that occurred within foraging groups are included. Limited connectivity for some nonfocal males likely reflects low sample sizes for those individuals.

**Fig. 2.**
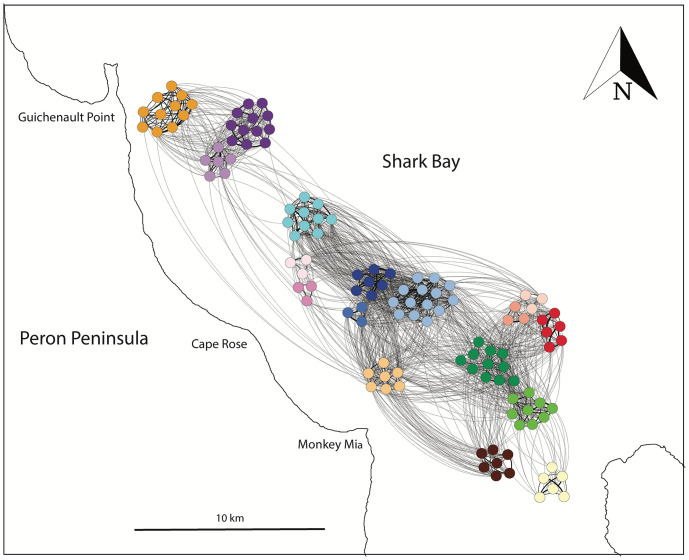
Social network plot of 12 second-order alliances and five trios (121 males). Edge weights represent association strength (i.e., social bond strength) calculated using the simple ratio index (SRI). Associations that occurred within foraging groups are excluded. The location of the second-order alliances are geo-referenced based on their approximate mating season core home range (see 14). Node colors denote alliance membership (second-order alliance or trio), with third-order allies (Table 1) sharing similar colors (from *top left* to *bottom right*: orange = SJ, dark purple = PB and purple = HH [third-order], turquoise = WC, light pink = BB and dark pink = SK [third-order], dark blue = PD and medium blue = RHP [third-order], light blue = KS [third-order with PD], light orange = RR, light pink = FCB and red = CB [third-order], medium pink = PHG [third-order with CB], dark green = BL and medium green = XF [third-order], mahogany = HC, yellow = GG).

The 121 focal males in this study resided in a continuous (or “connected”) social network where every male was connected directly or indirectly (Figs. 1 and 2). Within this network of 121 males, the mean number of adult males that each male directly associated with was 22, with a maximum of 50. For this calculation, we removed all associations that occurred when animals were foraging, because animals tend to loosely aggregate in large groups at the same foraging patch but are not necessarily associating preferentially. When including all foraging groups and all nonfocal adult males that were members of other alliances and present in the study area over the same period (*n* = 81), the average number of direct male associates for the focal males increased to 40, with a maximum of 76. These values are much larger than the average number of second- and third-order alliance relationships for the 121 males (average: 13, range: 5–23).

Among the 12 second-order alliances and five trios, we found considerable variation in both alliance size and the level of differentiation in both the strength of association and consortship relationships (Table 1). However, association and consortship relationships were highly correlated across alliances, with males tending to consort females with those males from their second-order alliance with whom they spend more time (MCMCglmm: 0.75, CI = 0.74–0.76, *n* = 121 _males_, *n* = 17 _alliances_, *P* = < 0.001).

**Table 1.** Measures of bond strength based on associations and consortships for 12 second-order alliances and five trios (total of 117 males*), where alliance ID and size are provided alongside the coefficient of variation (CV) of within-alliance dyadic relationship strength based on association data (calculated using the simple ratio index [SRI] after removing all foraging groups and surveys where a consortship occurred to avoid any overlap between our association and consortship measures) and the CV of within-alliance dyadic relationship strength based on consortship data.

Alliance ID	Size	CV of dyadic association SRI	CV of dyadic consortship SRI	Third-order allies
KS second-order	14	0.67	1.74	PD
PB second-order	12	1.95	1.89	HH
SJ second-order	11	0.62	1.75	
WC second-order	10	0.85	1.50	
BL second-order^†^	8	0.61	1.31	XF
XF second-order	8	0.81	1.53	BL
PD second-order	7	0.67	1.60	RHP, KS
RR second-order^†^	7	0.32	0.53	
HC second-order	7	0.62	2.04	
GG second-order	6	1.02	1.39	
CB second-order	6	0.83	1.26	FCB, PHG
HH second-order	6	0.56	1.09	PB
SK trio	3	0.08	0.24	BB
BB trio	3	0.17	0.13	SK
RHP trio	3	0.21	0.15	PD
FCB trio	3	0.21	0.31	CB
PHG trio	3	0.66	0.43	CB

Third-order alliances were determined by testing for between-alliance preferences using permutation tests in SOCPROG (26), as per Connor et al. (29). Average-linkage hierarchical clustering diagrams for each third-order alliance are provided in the *SI Appendix* (*SI Appendix*, Fig. S2).

^*^Four males are shown in Fig. 2 but are not included in our main analyses as they either never successfully joined the alliance (one male in the XF alliance) or joined the alliance toward the end of our study period (2006; three males in the BL alliance).

^†^Alliances that matured during the 2001–2006 study.

**Fig. 3.**
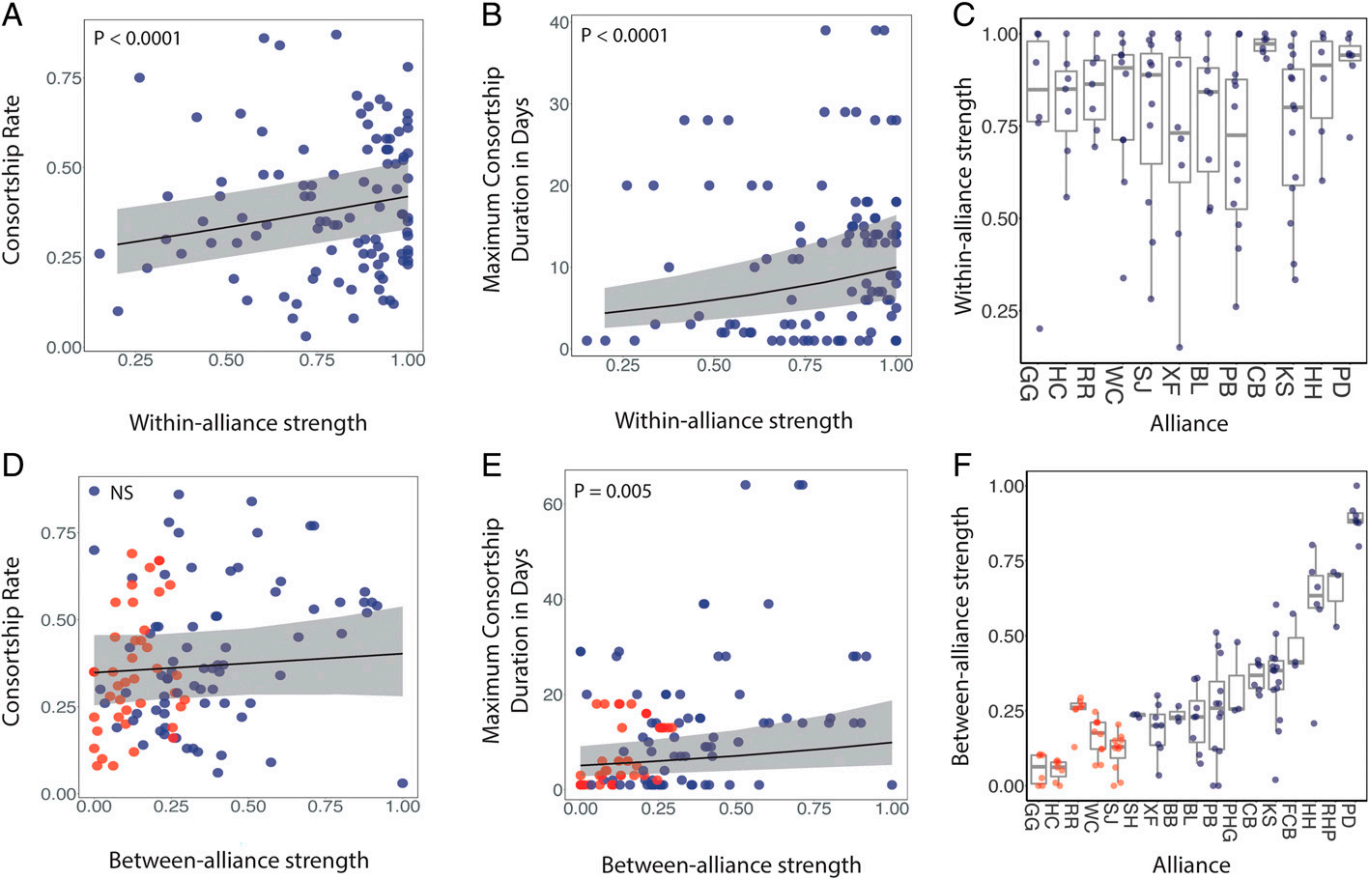
Relationship between normalized cumulative social bond strength and consortship success. (*A*) Consortship rate (*n* = 102 males) and (*B*) maximum consortship duration (*n* = 102 males) within second-order alliances as a function of normalized cumulative strength of social bonds with second-order allies when not consorting females (normalized for second-order alliance size). (*C*) Boxplots of normalized cumulative social bond strength for individual males within each second-order alliance. (*D*) Consortship rate (*n* = 117 males) and (*E*) maximum consortship duration (*n* = 117 males) between alliances as a function of normalized cumulative strength of social bonds with males outside the second-order alliance (normalized for network size). (*F*) Boxplots of normalized cumulative social bond strength with males outside the second-order alliance (i.e., between-alliance bond strength) for individual males within each second-order alliance. In panels (*D*–*F*), red points represent males that do not have third-order allies and blue points represent males that do have third-order allies (Table 1). All panels show raw data (blue or red points) with model estimates (solid line) and 95% CIs (shared area) in panels (*A* and *B*) and (*D* and *E*).

When restricting our analyses to second-order alliances only, without including trios, the cumulative strength of social bonds with second-order allies when not consorting females (normalized for second-order alliance size) predicted consortship rate (days observed in consortships/total days observed) within second-order alliances (glmer estimate: 0.73, CI = 0.38–1.09, *z =* 4.12, *n* = 102 _males_, *n* = 12 _alliances_, *P* < 0.0001, Fig. 3*A*), as well as maximum consortship duration (length in days of the longest consortship in which a male participated) within second-order alliances (glmer estimate: 1.03, CI = 0.71–1.35, *z =* 6.28, *n* = 102 _males_, *n* = 12 _alliances_, *P* < 0.0001, Fig. 3*B*), whereas, in the same models, second-order alliance size did not significantly predict consortship rate (glmer estimate: 0.10, CI = −0.04–0.25, *z =* 1.44, *n* = 102 _males_, *n* = 12 _alliances_, *P* = 0.14; *SI Appendix*, Fig. S3*A*) or consortship duration (glmer estimate: 0.09, CI = −0.09–0.29, *z =* 1.07, *n* = 102 _males_, *n* = 12 _alliances_, *P* = 0.28; *SI Appendix*, Fig. S3*B*). We included consortship duration as a measure because many consortships last a day or less, particularly if males are unable to defend the female from attacks from rivals or the female is not maximally attractive (*SI Appendix*). Some males can successfully defend a female from rivals and keep her for extended periods of time. Given that consortship durations were highly skewed, with many observed on 1 d only, we used maximum consortship duration as a measure of the potential of an individual to keep a female. These findings indicate that males who were well connected in their second-order alliances (Fig. 3*C*), irrespective of alliance size, tended to spend more time consorting females (Fig. 3*A*) and consorted individual females for longer (Fig. 3*B*).

We then investigated whether between-alliance social relationships increased access to females but this time using association data from the five trios, as well as the 12 second-order alliances. We found that the cumulative strength of social bonds with males outside the second-order alliance (normalized for network size) did not predict consortship rate (glmer estimate: 0.23, CI = −0.34–0.80, *z =* 0.79, *n* = 117 _males_, *n* = 17 _alliances_, *P* = 0.42; Fig. 3*D*) but did significantly predict consortship duration (glmer estimate: 0.66, CI = 0.20–1.13, *z =* 2.80, *n* = 117 _males_, *n* = 17 _alliances_, *P* = 0.005; Fig. 3*E*), whereas, in the same models, third-order alliance size did not significantly predict consortship rate (glmer estimate: 0.04, CI = −0.02–0.11, *z =* 1.37, *n* = 117 _males_, *n* = 17 _alliances_, *P* = 0.17) or consortship duration (glmer estimate: 0.08, CI = −0.01–0.18, *z =* 1.76, *n* = 117 _males_, *n* = 17 _alliances_, *P* = 0.07). This suggests that males forming stronger relationships with other males outside their second-order alliance, i.e., third-order alliances (Table 1 and Figs. 2 and 3*F*), can consort females for significantly longer, independently of third-order alliance size.

The aforementioned models were run separately for within-alliance and between-alliance relationships due to differing sample sizes. However, we also reran the models on a subset of data that included both the cumulative strength of social bonds with second-order allies and the cumulative strength of social bonds with males outside the second-order alliance, as well as second- and third-order alliance size, to determine whether these variables independently influenced consortship behavior. Within-alliance social ties still significantly predicted consortship rate (within-alliance social ties, *P* < 0.0001; between-alliance, *P* = 0.91; *n* = 102 _males_, *n* = 12 _alliances_; *SI Appendix*, Table S1), and both within- and between-alliance social ties still significantly predicted maximum consortship duration (within-alliance, *P* = < 0.0001; between-alliance, *P* = 0.007; *n* = 102 _males_, *n* = 12 _alliances_; *SI Appendix*, Table S1), whereas alliance size had no effect on either response variable (*SI Appendix*, Table S1).

## Discussion

The 200+ male dolphins, and the 121 focal males that formed the core of this study, represent the largest alliance network known in any nonhuman species (*SI Appendix*). We have shown the dolphin alliance social network is continuous and that individual males socialize with numerous males outside of their own three-level alliance network. Within second-order alliances, the core social unit of male social organization, males consort females with males with whom they share a stronger social bond and males that are more socially connected within their second-order alliance have more success consorting females. This is expected if variation in alliance bonds reflects the outcome of strategic social decisions. Finally, we have shown the importance of third-order alliance relationships (those between members of different second-order alliances) for male reproductive success: males that are more socially connected with third-order allies have more success consorting females. Below, we examine these outcomes in more detail and compare them to alliance formation in humans and other species.

We demonstrated previously that the Shark Bay dolphin society is “open”, with no alliances or combination of alliances patrolling and defending the entire or specific areas (14). Rather, there is a continuous mosaic of male alliance and individual female home ranges. However, even with range overlap, a male’s alliance network might be a closed social unit, like those of primate bisexual groups with broadly overlapping ranges (e.g., (36)). Here, using conservative criteria that eliminate sightings where individuals are together because they are merely attracted to the same foraging resource, we have shown that the male alliance network is not closed but socially continuous. All 121 males are connected directly or indirectly in social groups in the largest alliance network known outside of humans. On average, each male was found in social groups with 22 of the 121 focal males (maximum 50). Less conservatively, we calculated males’ “social exposure” to other males using all 202 males (121 focal and 81 nonfocal males) in all groups, including foraging groups, where, on average, each male was exposed to 40 other males (maximum 76). It must be stressed that both these values will be underestimates, as we observe individual males for such a small portion of their lives.

Notably, each male’s total number of male associates is considerably larger than the number of second- and third-order alliance relationships he maintains (average: 13, range: 5–23), revealing that male-male social networks are not closed structures constrained by the size of their second- and third-order alliances. Individual males may expand their strategic options by maintaining social familiarity with males outside of their three-level alliance network, possibly leading to the formation of new alliance relationships, as occurs, for example, when males occasionally join an existing alliance after a member disappears ((22), *SI Appendix*).

We examined how the cumulative strength of social bonds with second-order allies impacted a male’s reproductive success. Here, we found that how well connected a male was in his second-order alliance predicted consortship rate and maximum consortship duration. We have previously shown that males who have stronger bonds within their second-order alliance secure more paternities (25). Here, we show that this is likely explained by the fact that socially integrated males within second-order alliances spend more time consorting females and can consort them for longer. Indeed, the rate at which males consort females varies considerably within second-order alliances and is related to alliance stability (22, 23), as well as cumulative social bond strength with second-order allies, as shown here. Independently, how well connected a male was outside of his second-order alliance (with his third-order alliance partners) also predicted maximum consortship duration, with longer-duration consortships providing males with an increased opportunity to copulate with females and secure paternities. This result supports our hypothesis that third-order alliance relationships provide males with additional “insurance” to defend females from theft attempts by rival males, especially in situations where second-order allies are not nearby (22, 29). Thus, each male navigates a multilevel alliance network of strongly differentiated social relationships that includes cooperation and competition at all levels (22, 24, 29).

Males form alliances with unrelated peers of similar ages with whom they associated during the juvenile period (27, 28). We did not include male age in our analyses because age is a significant predictor of alliance formation, i.e., second-order allies tend to be of a similar age (27), age does not influence a male’s number of paternities (25), and mature males of all ages participate in third-order alliances (24, 29). We have suggested that alliance partners are selected strategically based on social and ecological homophily, because alliances with broadly overlapping ranges differ in preferred foraging habitat and behavior ((37, 38), see *SI Appendix*). Our finding that second-order alliance size is unrelated to consortship rate and duration is unsurprising if second-order alliance size is related to alliance variation in foraging behavior (38) and perhaps demographic variation in the availability of potential partners. However, we have shown that the strategic investment in third-order alliance relationships does increase access to a contested resource, through an increase in the duration of consortships.

It therefore appears that the Shark Bay dolphins have converged with humans and one of our nearest relatives, chimpanzees, in remarkable but different ways (21, 22, 28, 39). With chimpanzees, the dolphins share general life history traits, a fission-fusion grouping pattern, large testes and a promiscuous mating system that includes aggressively formed consortships with individual females, and strategic within-group male coalitions and alliances (12). As in chimpanzees, bond strength among male dolphins is directly associated with access to females (40, 41). The coalitions formed by male chimpanzees to increase rank are famously strategic, as individuals may opportunistically “switch sides” (31, 42), but interactions between males from different communities are exclusively hostile (43, 44).

With humans, but not chimpanzees, Shark Bay dolphins share two characteristics: extremely large brains that are three times larger than similar-sized relatives (45, 46) and the formation of strategic, multilevel male alliances (33, 45). While these dolphins reside in a truly open society, unlike chimpanzees and humans, humans effectively achieve an open social network because they maintain relations with dispersed relatives and are thus “released from the constraint of proximity” (1). It appears likely that the most recent common ancestor of humans and chimpanzees was more similar to the common chimpanzee than other apes (47–49). Human multilevel alliances occur with other divergent traits, such as the ubiquitous pair bond and allocare, including male parental investment. Current models of human social evolution link intergroup alliances directly or indirectly with these other traits (2, 4, 34, 35, 50–52), possibly through a transitional multilevel social structure formed around one-male units, as found in hamadryas (*Papio hamadryas*) and guinea baboons (*P. papio*) (2, 8, 53, 54). Our study shows that strategic, intergroup male alliances can arise directly from a chimpanzee-like promiscuous mating system without one-male units, pair bonds, or male parental care.

Our results bear on the “social brain” hypothesis for the evolution of large brains and intelligence, which holds that complex social relationships were the key driver in the evolution of large brains and intelligence (5, 55, 56). Complex social relationships are exemplified by coalitions and alliances within social groups (57), where alliance relationships are cultivated with affiliative interactions, often between nonrelatives, with the potential for individuals to switch sides opportunistically (e.g., (31, 42)). Increasing the number of alliance levels will also increase the cognitive demands of alliance formation, as decisions at one level may impact success at another level (33).

Male dolphin relationships are strongly differentiated, not only between but within each alliance level (22–24, 32, 33). The number and types of differentiated relationships impact selection for enhanced social cognition to the extent that they include options and risk, whereby males have options to choose different allies, but those choices entail an element of risk due to their fitness consequences (33). The dolphin alliances conform to expectations of a system based on choice and competition for allies, as males form alliances primarily with unrelated age mates (27, 28) and context-dependent interactions occur at all three levels (22, 29, 58). The risk entailed by male dolphins’ alliance options are represented here by our finding that bonds with second- and third-order allies impact male consortship success. Multilevel strategic alliances are a prominent feature of human societies; thus, the discovery of strategic, multilevel male alliances in dolphins is a surprising case of convergence that broadens our understanding of human social and cognitive evolution.

## Materials and Methods

Data were collected from 2001 to 2006 in the eastern gulf of Shark Bay, Western Australia, where our research on Indo-Pacific bottlenose dolphins has been carried out on a near-annual, seasonal basis (typically austral winter and spring) since 1982 (22, 59). Data on associations and consortships were collected on 202 males, but subsequent analyses focused on the most frequently observed 121 “focal” males. Extensive consortship data had been collected on these males, who were members of 12 second-order alliances and five trios, which were the remnants of formerly larger second-order alliances (29). Of the focal males, one (FAR) never successfully joined the XF alliance and three (TOL, POO, and HHD) joined the BL alliance toward the end of our study period (2006). They were, therefore, not included in our main analyses but are included in Figs. 1 and 2. At least seven second-order alliances with poorly established membership (one large putative second-order alliance might have been two) and few consortship records were excluded from the focal male analysis.

Association data were collected during boat-based surveys between June and December each year, which includes the peak mating season (September to November). A survey is a minimum 5-min snapshot of dolphin group composition (as defined by the 10 m chain rule, where each dolphin in the group is within 10 m of any other dolphin (13)) and behavioral activity. Only association data recorded in the first 5 min of a survey were used to ensure association measures were comparable across surveys. Resights, where the same group is encountered within 2 h, were excluded.

We constructed a social network from surveys to determine whether the social network among the 121 focal males is continuous (each male is at least indirectly socially connected to every other male) or discontinuous (all males are not indirectly connected). We removed all foraging surveys (defined based on interindividual spacing, relative orientation, dive type, and direct observations of prey or feeding), as animals tend to loosely aggregate in large groups at the same foraging patch but are not necessarily associating preferentially, and we removed surveys in which a fight was occurring between two or more alliances in the first 5 min (two such cases). We then calculated the number of connections for each focal male, i.e., the number of males in each focal male’s social network. To assess each of the 121 focal males’ broader social exposure to other males, we also calculated each focal male’s social network size inclusive of all 202 males, including foraging groups.

For reasons explained above and prior to calculating association indices, we removed all foraging groups and surveys where a consortship occurred to avoid any overlap between our association measures and measures of consortship behavior (see below). We calculated association indices using the simple ratio index (SRI) (60, 61) in the R package *asnipe* (62), which is an estimate of the proportion of time two animals spend together (0 for pairs of animals never observed together; 1 for pairs always seen together). Given the high degree of fission-fusion dynamics in bottlenose dolphin societies, association indices are a measure of bond strength and reflect true social preferences, i.e., individuals have more choice of associates than those living in relatively stable social groups (63). We then used the R package *sna* (64) to calculate “strength”, i.e., cumulative strength of social bonds, for within second-order alliances and between alliances, i.e., relationships between males in different second-order alliances/trios.

For our measures of consortship behavior, we used all consortships that occurred between June and December each year. Each consortship was used once, and resights were excluded. We calculated the strength of consortship relationships using the SRI (0 for pairs of animals that never consort females together; 1 for pairs that always consort females together). For each male, we calculated their consortship rate as the number of days observed in a consortship divided by the total number of days observed (following (23, 65)) and their maximum consortship duration, which is the span in days from the first to the last day males were sighted in a consortship with a particular female. Except in cases where we see the female captured, escape, or taken by rivals, consortship durations are conservative, given that we are unable to monitor males every day.

### Statistical Analysis.

All statistical procedures were conducted in R 4.0.2 (66). To measure the differentiation in both the association and consortship relationships, we calculated the coefficient of variation (CV) of both the association and consortship relationship measures using the SRI within each alliance (Table 1). To check for correlation between the two datasets, we ran a multimembership linear model that includes a node dependence term in MCMCglmm (67), where the response variable was the consortship SRI (dyadic relationship strength based on consortship data) and the predictor variable was the association SRI (dyadic relationship strength based on association data). Each node was included as a random effect using the multimembership function in R.

To determine whether the strength of a male’s cumulative social ties within the second-order alliance influenced their consortship behavior, we calculated node strength (cumulative strength of social bonds) from the association data for each male within each alliance. We restricted this analysis to second-order alliances only (i.e., we excluded the trios). We normalized the strength values for each alliance to compare relative strength values across different-sized second-order alliances. This was achieved by dividing each male’s strength by the maximum strength value in that alliance, thus scaling node strength between 0 and 1 for each alliance. We also calculated cumulative strength of social bonds with males outside the second-order alliance only (i.e., third-order allies) only for each focal male using data from all 12 second-order alliances and the five trios. We normalized this value by dividing each males’ between-alliance strength by the maximum between-alliance strength value, thus scaling between-alliance strength between 0 and 1 for all males.

To determine how cumulative social bond strength with second-order allies predicted a male’s consortship rate or his maximum consortship duration, we built a generalized linear mixed-effect model with binomial family for proportion data, where consortship rate was the response variable, and a generalized linear mixed-effect model with a Poisson family for count data (*lme4* package in R (68)), where maximum consortship duration (in days) was the response variable. For these analyses, we only included data from the 12 second-order alliances and not the five trios. For both models, the fixed effects were normalized cumulative social bond strength with second-order allies and second-order alliance size. Alliance ID was included as a random effect. We then investigated whether cumulative social bond strength with males outside the second-order alliance, i.e., between-alliance relationships, influenced a male’s consortship rate or his maximum consortship duration. For these analyses, we included data from all 12 second-order alliances and five trios. We built a generalized linear mixed-effect model with binomial family for proportion data, where consortship rate was the response variable, and a generalized linear mixed-effect model with a Poisson family for count data, where maximum consortship duration (in days) was the response variable. For both models, our fixed effects were normalized cumulative social bond strength with males outside of the second-order alliance and third-order alliance size. Alliance ID was included as a random effect. For each model, we employed a traditional hypothesis testing approach, where we used ANOVA (*car* package in R (69)) to test whether the full model explained significantly more variance than the null model (model without our fixed effect of interest, i.e., cumulative social bond strength). We used the *DHARMa* package (70) to assess model fit, and we used the *effects* (69) and *ggplot2* (71) packages in R to plot model estimates over the raw data.

Finally, to confirm that within-alliance and between-alliance relationships were influencing consortship behavior independently, we ran two further generalized mixed-effect models for consortship rate (binomial family for proportion data) and consortship duration (Poisson family for count data) with normalized cumulative strength of social bonds with second-order allies, normalized cumulative strength of social bonds with males outside the second-order alliance, second-order alliance size and third-order alliance size as fixed effects, and alliance ID as a random effect. We calculated the variance inflation factor for each fixed effect to check for collinearity, and all values were ≤ 3 (72) and were thus retained in the model. We were only able to do this for the 12 second-order alliances where cumulative social bond strength within- and between-alliances was available, i.e., the trios were not included in this follow-up analysis.

## Supplementary Material

Supplementary File

## Data Availability

All data are included in the manuscript and/or supporting information.
