# Supplementary material for: Strategic intergroup alliances increase access to a contested resource in male bottlenose dolphins
Source: Proc Natl Acad Sci U S A. 2022 Aug 29;119(36):e2121723119. doi: 10.1073/pnas.2121723119 (PMC9457541; doi:10.1073/pnas.2121723119)
Supplement: Supplementary File [file pnas.2121723119.sapp.pdf]

SI APPENDIX

Strategic intergroup alliances increase access to a contested resource in male bottlenose dolphins

Richard C. Connor [1,2Ψ*], Michael Krützen [3], Simon J. Allen [3,4,5], William B. Sherwin [6] and Stephanie L. King [4,5Ψ*]

[1] Biology Department, University of Massachusetts Dartmouth, North Dartmouth, MA 02747, USA
[2] Department of Biological Sciences, Marine Sciences Program, Florida International University, 3000 NE 151st St., North Miami, FL 33181, USA
[3] Evolutionary Genetics Group, Department of Anthropology, University of Zurich, CH-8057 Zurich, Switzerland
[4] School of Biological Sciences, University of Bristol, Bristol, BS8 1TQ, United Kingdom
[5] School of Biological Sciences, University of Western Australia, Crawley, WA 6009, Australia
[6] Evolution and Ecology Research Centre, School of Biological, Earth and Environmental Sciences, UNSW, Sydney, NSW 2052, Australia

[Ψ] These authors contributed equally to this work.

* Correspondence to: Richard Connor, Biology Department, University of Massachusetts Dartmouth, North Dartmouth, MA 02747, USA, rconnor@umassd.edu, and Stephanie King, School of Biological Sciences, University of Bristol, Bristol, BS8 1TQ, UK; stephanie.king@bristol.ac.uk; ORCID: 0000-0003-2293-9185

The Shark Bay dolphin society: habitat and alliance behavior

The dolphins' habitat is quite diverse, including shallows that grade into open embayment plains and deeper channels bounded by steep slopes rising to shallow seagrass covered banks. The dolphins pursue a wide variety of prey in all of these habitats. Male alliances specialize in foraging habitats and, in one case, foraging behavior (1, 2). Further, there is systematic variation in alliance behavior along the 50km length of the study site that suggests increasingly marginal habitat in the southern, higher salinity, zone (see (3)). Specifically, males form more trios vs pairs in the north (which may also reflect communication distance in open water), consort females at a higher rate, and suffer more fresh bite marks indicating conflicts with rivals (4, 5).

Operational definition of alliances

De Waal & Harcourt (6) defined a coalition as "the joining of forces by two or more parties during a conflict of interest with other parties" and an alliance as "enduring cooperative relationships. So an alliance manifests itself in repeated coalition formation between two individuals, but not every coalition needs to reflect an alliance."

While this clearly applies to our second- and third-order alliances, this coalition/alliance dichotomy cannot be applied to the dolphin first-order alliances because the stability of first-order alliances lies on a continuum, with some males forming highly stable trios and others participating in trios or pairs of variable composition. This is true whether we consider the stability of each male's first-order trio or choice of a particular partner in that trio (see the distribution of values on the x axis in Fig. 3 in Connor et al. (7) and Connor and Krützen (8). Further, a male might form the same trio to consort females 'repeatedly' but consort females with other combinations of males between those events. Given the dolphin trio composition continuum, we made a decision early in the study to use one term, 'alliance' for cases of cooperation against conspecifics, irrespective of the extent to which a particular combination of males is repeated. We have made this point before, but in the ESM of papers. For example, Connor et al. ((9), ESM):

Use of the term 'alliance' vs 'coalition'. Some authors have advocated the use of the term 'coalition' for instances of cooperation against conspecifics and 'alliance' for more enduring relationships with

repeated instances of cooperation (e.g. Harcourt & DeWaal 1992). This dichotomy is not useful for our dolphin study as there is a continuum from a particular combination of 2-3 males observed consorting together only once to long-term highly stable associations over many consortships and years.

Consorting females (i.e., forming first-order alliances) with third-order allies is rare

Connor et al. (9) reported that, of 476 consortships by second-order alliance members, 15 (3%) contained non-members. Here, we further examine these 15 'anomalous' cases. Nine of the 15 cases were consortships with males that were not third-order allies, but with four males that were not members of a second-order alliance (we suspect that such consortships are a way for males to attempt to join alliances (8)). In the seven third-order alliances identified based on association permutation tests, only two had any mixed alliance level consortships. In one third-order alliance, a male herded with a pair from the other second-order alliance on one occasion (of 167 consortships by the two second-order alliances). The case may have owed to the male's lack of opportunity in his second-order alliance, where he was in only one of 59 consortships. In a different third-order alliance, one male from each second-order alliance herded with a pair of males from the other second-order alliance once and four times, respectively (totaling five out of 73 consortships). The male in the single case participated in 14 of 26 consortships in his second-order alliance of six individuals that became five after one member disappeared. That disappearance from an alliance that consorted females in trios only (see (4)) may have precipitated their third-order ally consorting with them four times, because his inclusion allowed them to form two trios again. That male also continued to associate and consort females in his 'home' second-order alliance. In sum, these six cases constitute just 1.5% of the 388 consortships by members of the seven third-order alliances.

Second-order alliance membership is largely closed

Second-order alliances are formed by mostly unrelated, similar-aged males in their teens (10, 11). The actual pattern by which alliances form may vary; we have observed one alliance of seven 'crystalize' over a three-year period as associations increased, and watched as a young second-order alliance of eight grew to 14 over a six-year period as newly maturing males in the area continued to join (8). However, we have recorded only six cases where a mature male left one second-order alliance and joined another

group of males. Two of these cases were apparent evictions, coinciding with the disappearance of a key ally in the group, and resulted in the males joining groups of immature males that were not yet consorting females. When those groups matured and began consorting females, the older males were able to consort females again. In one case, a central member of a group of 14 left his second-order alliance for another group. The remaining three cases were old males who were the last survivors of their second-order alliance and were able to join new groups. In two cases, they joined second-order alliances and, in the other case, due to a gap in observations, it was unclear if he joined a second-order alliance or an immature group (8). Two of the six transfers occurred in the second-order alliance with the longest study history (the WC group, (12)), who were first documented in late 1994 as a group of 14. Sometime between 1997 and 2001, a central member moved to another second-order alliance, where he was observed consorting females from 2001 onward. By 2009, the WC group had diminished to eight individuals which left two males unable to consort females since they strongly preferred trios. All members were able to consort females after the lone survivor of rival second-order alliance joined them to form a new trio in 2011 (see details in (8)).

Since the Shark Bay bottlenose dolphins live in an open society with bisexual philopatry, there is no natal dispersal from one 'semi-closed' group to another, as occurs in primates and many other terrestrial mammals. The unusual cases of adult male transfer that we have observed between dolphin second-order alliances would be more nearly equivalent to secondary transfers in primate groups (e.g., (13–15)). The bisexual philopatry of many odontocetes and the open society of the Shark Bay dolphins likely relate to very low costs of locomotion that dolphins experience at slow speeds. This allows for day ranges that exceed that of all terrestrial mammals by an order of magnitude and large home ranges that enable dolphins to interact with many non-relatives (16) .

Consortship duration

The duration of consortships is highly skewed; a large percentage of consortships are observed on one day only (17). The short duration of many consortships may owe to the inability of males to defend females, the females not being maximally attractive and, in a few cases males may herd females to reinforce male-male bonds (17, 18).

For reasons that are not completely understood, but may have to do to paternity confusion to reduce infanticide risk, females that conceive during the breeding season (September-December, calculated from offspring birth assuming a 12-month gestation) are often consorted during pre-breeding season months (July-August) (17). Such pre-breeding season consortships are typically brief (≤ 1day) although a few females conceive and are engaged in long-duration consortships outside of the breeding season (17). Consortships are longer during the breeding season when most conceptions occur but males will have greater difficulty keeping females during this time due to intense competition from other alliances.

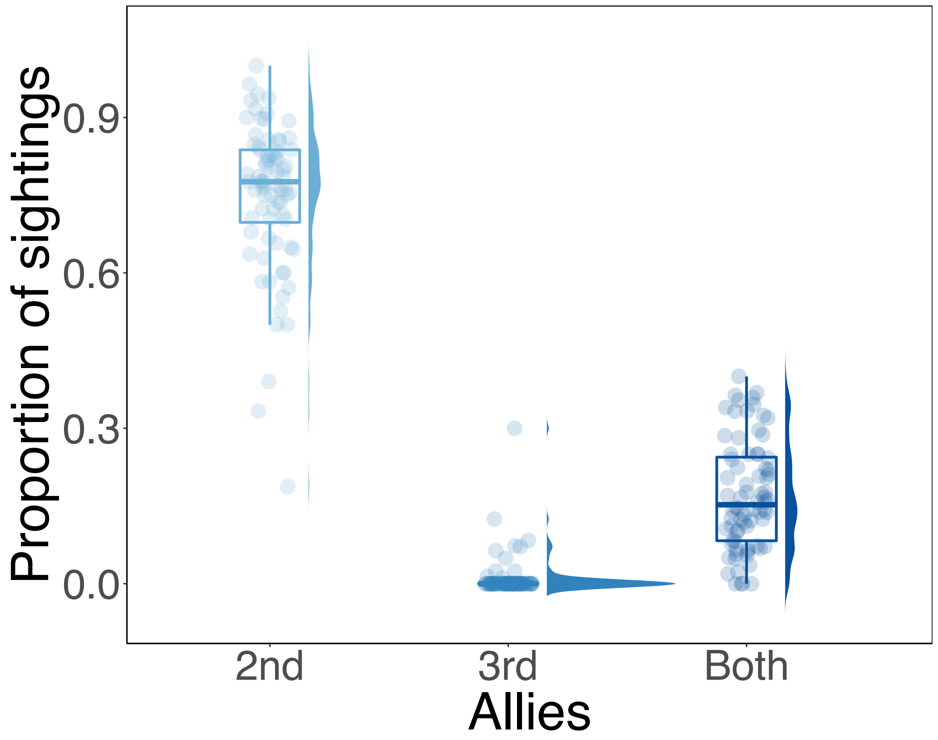

Figure S1. Proportion of sightings with second- and third-order allies for each male within a third-order alliance. The proportion of total sightings where males are seen with just their second-order allies (light blue), just their third-order allies (medium blue), or with members of both their second- and third-order alliances (dark blue). Filled circles represent raw data.

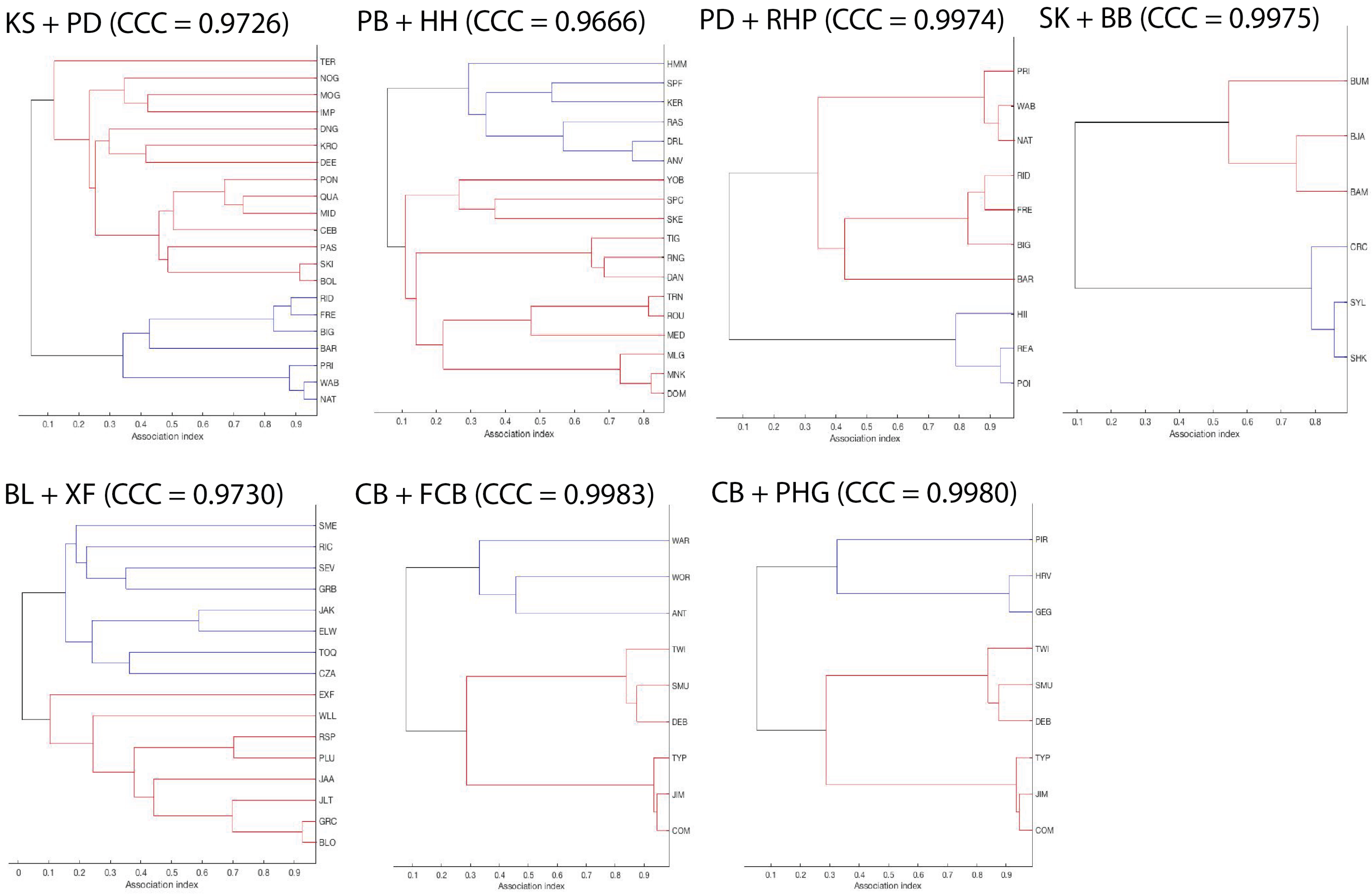

Figure S2. Third-order alliance relationships. Average-linkage cluster diagram, based on association indices (Simple Ratio Index) among the seven third-order alliances from 2001-2006. The two-letter codes of each second-order alliance are presented above each diagram with the cophenetic correlation coefficient (CCC), where a CCC > 0.8 indicates that the hierarchical model provides a good representation of the social network (19). Each diagram shows the three-letter male ID code and each second-order alliance is color-coded (red and blue).

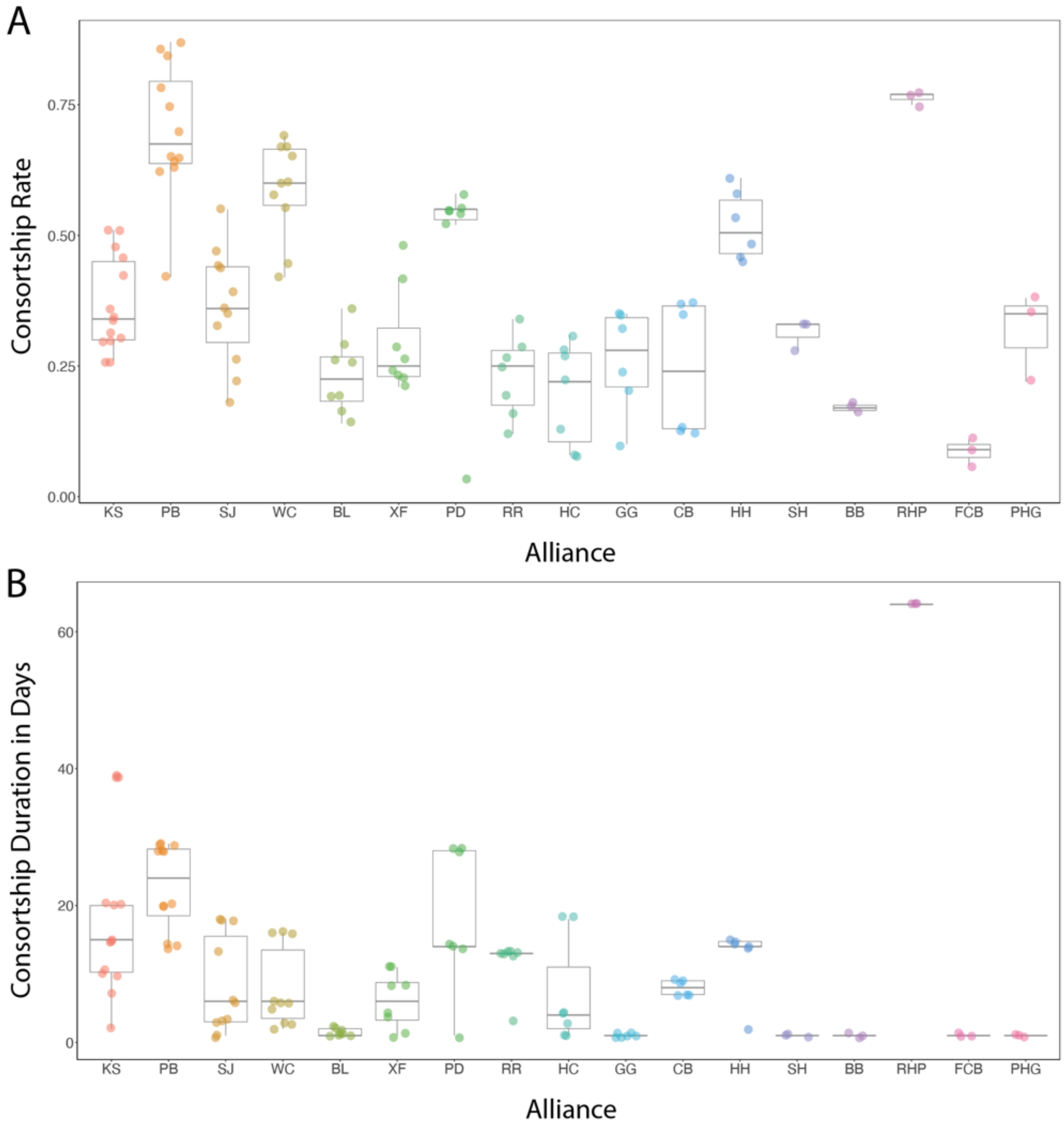

Figure S3. Consortship rate and maximum observed consortship duration for individual males within each alliance. Alliances are ordered by size (Table 1).

Table S1. Parameter estimates for generalized mixed-effect models for consortship rate (binomial family for proportion data) and consortship duration (Poisson family for count data) as a function of normalized cumulative strength of social bonds with second-order allies ('second-order ties'), normalized cumulative strength of social bonds with males outside the second-order alliance ('third-order ties'), second-order alliance size and third-order alliance size. Alliance ID was included as a random effect. Data based on 102 males from 12 alliances. Variables were untransformed in the models, however, centering variables with a mean of 0 and an SD of 0.5 had little to no effect on the test statistic and p-values and did not change the results.

(i) Consortship Rate	Estimate	SE	Confidence Interval	*z* value	*p* value
Intercept	-2.23	0.69	-3.69 – -0.78	-3.2	**0.001**
within alliance ties	0.73	0.18	0.37 – 1.09	4.0	**< 0.0001**
between alliance ties	0.03	0.32	-0.59 – 0.66	0.1	0.91
second-order size	0.07	0.07	-0.08 – 0.24	1.0	0.31
third-order size	0.03	0.03	-0.04 – 0.10	0.8	0.38
(ii) Consortship duration					
Intercept	-0.08	0.80	-1.81 – 1.61	-0.10	0.91
within alliance ties	1.01	1.65	0.69 – 1.34	6.12	**< 0.0001**
between alliance ties	0.71	0.26	0.19 – 1.23	2.68	**0.007**
second-order size	0.09	0.08	-0.10 – 0.28	1.04	0.29
third-order size	0.01	0.03	-0.06 – 0.10	0.49	0.61

p-values in bold indicate significant results.

References

1. O. O'Brien, S. J. Allen, M. Krützen, R. C. Connor, Alliance-specific habitat selection by male Indo-Pacific bottlenose dolphins in Shark Bay, Western Australia. *Anim. Behav.* **164**, 39–49 (2020).
2. M. R. Bizzozzero, *et al.*, Tool use and social homophily among male bottlenose dolphins. *Proc. R. Soc. B Biol. Sci.* (2019) https:/doi.org/https://doi.org/10.1098/rspb.2019.0898.
3. B. W. Logan, D. E. Cebulski, "Sedimentary environments of Shark Bay, Western Australia" in *Carbonate Sedimentation and Environments, Shark Bay, Western Australia*, B. W. Logan, G. R. Davies, J. R. Read, D. E. Cebulski, Eds. (The American Association of Petroleum Geologists, 1970), pp. 1–37.
4. R. C. Connor, *et al.*, Male alliance behaviour and mating access varies with habitat in a dolphin social network. *Sci. Rep.* **7** (2017).
5. R. A. Hamilton, *et al.*, Male aggression varies with consortship rate and habitat in a dolphin social network. *Behav. Ecol. Sociobiol.* **73**, 141 (2019).
6. F. B. M. de Waal, A. H. Harcourt, "Coalitions and alliances: a history of ethological research" in *Coalitions and Alliances in Humans and Other Animals*, (Oxford University Press, 1992), pp. 3–19.
7. R. C. Connor, M. R. Heithaus, L. M. Barre, Complex social structure, alliance stability and mating access in a bottlenose dolphin "super-alliance". *Proc. Biol. Sci.* **268**, 263–267 (2001).
8. R. C. Connor, M. Krützen, Male dolphin alliances in Shark Bay: Changing perspectives in a 30-year study. *Anim. Behav.* **103**, 223–235 (2015).
9. R. C. Connor, J. J. Watson-Capps, W. B. Sherwin, M. Krützen, A new level of complexity in the male alliance networks of Indian Ocean bottlenose dolphins (*Tursiops* sp.). *Biol. Lett.* **7**, 623–626 (2011).
10. L. Gerber, *et al.*, Affiliation history and age similarity predict alliance formation in adult male bottlenose dolphins. *Behav. Ecol.* **31**, 361–370 (2020).
11. L. Gerber, *et al.*, Cooperative partner choice in multi-level male dolphin alliances. *Sci. Rep.* **11**, 1–10 (2021).
12. R. C. Connor, M. R. Heithaus, L. M. Barre, Superalliance of bottlenose dolphins. *Nature* **397**, 571–572 (1999).
13. M. R. Clarke, K. E. Glander, Secondary transfer of adult mantled howlers (*Alouatta palliata*) on Hacienda La Pacifica, Costa Rica: 1975–2009. *Primates* **51**, 241–249 (2010).
14. K. M. Jack, L. M. Fedigan, Male dispersal patterns in white-faced capuchins, *Cebus capucinus* part 2: patterns and causes of natal emigration. *Anim. Behav.* **67**, 771–782 (2004).
15. S. Van Belle, A. Di Fiore, Dispersal patterns in black howler monkeys (*Alouatta pigra*): Integrating multiyear demographic and molecular data. *Mol. Ecol.* **31**, 391–406 (2022).
16. R. C. Connor, "Group living in whales and dolphins" in *Cetacean Societies: Field Studies of Whales and Dolphins*, J. Mann, R. C. Connor, P. L. Tyack, W. H., Eds. (University of Chicago Press, 2000), pp. 199–218.
17. R. C. Connor, A. F. Richards, R. A. Smolker, J. Mann, Patterns of Female Attractiveness in Indian Ocean Bottlenose Dolphins. *Behaviour* **133**, 37–69 (1996).
18. R. Connor, J. Mann, "Social Cognition in the wild: Machoavellian dolphins" in *Rational Animals*, (2006).
19. H. Whitehead, SOCPROG programs: analysing animal social structures. *Behav. Ecol. Sociobiol.* **63**, 765–778 (2009).
